# An Improved Lightweight Model for Protected Wildlife Detection in Camera Trap Images

**DOI:** 10.3390/s25237331

**Published:** 2025-12-02

**Authors:** Zengjie Du, Dasheng Wu, Qingqing Wen, Fengya Xu, Zhongbin Liu, Cheng Li, Ruikang Luo

**Affiliations:** 1 College of Mathematics and Computer Science, Zhejiang A&F University, Hangzhou 311300, China; duzengjie@stu.zafu.edu.cn (Z.D.);; 2 Key Laboratory of State Forestry and Grassland Administration on Forestry Sensing Technology and Intelligent Equipment, Hangzhou 311300, China; 3 Key Laboratory of Forestry Intelligent Monitoring and Information Technology of Zhejiang Province, Hangzhou 311300, China; 4 Wucheng Nanshan Provincial Nature Reserve Management Center of Zhejiang Province, Jinhua 321000, China

**Keywords:** lightweight deep learning, object detection, protected wildlife, camera traps, YOLO

## Abstract

Effective monitoring of protected wildlife is crucial for biodiversity conservation. While camera traps provide valuable data for ecological observation, existing deep learning models often suffer from low accuracy in detecting rare species and high computational costs, hindering their deployment on edge devices. To address these challenges, this study proposes YOLO11-APS, an improved lightweight model for protected wildlife detection. It enhances the YOLO11n by integrating the self-Attention and Convolution (ACmix) module, the Partial Convolution (PConv) module, and the SlimNeck paradigm. These improvements strengthen feature extraction under complex conditions while reducing computational costs. Experimental results demonstrate that YOLO11-APS achieves superior detection performance compared to the baseline model, attaining a precision of 92.7%, a recall of 87.0%, an mAP@0.5 of 92.6% and an mAP@0.5:0.95 of 62.2%. In terms of model lightweighting, YOLO11-APS reduces the number of parameters, floating-point operations, and model size by 10.1%, 11.1%, and 9.5%, respectively. YOLO11-APS achieves an optimal balance between accuracy and model complexity, outperforming existing mainstream lightweight detection models. Furthermore, tests on unseen wildlife data confirm its strong transferability and robustness. This work provides an efficient deep learning tool for automated wildlife monitoring in protected areas, facilitating the development of intelligent ecological sensing systems.

## 1. Introduction

As critical components of global biodiversity conservation, nature reserves play an essential role in sustaining protected wildlife and monitoring ecosystem dynamics [1]. According to the International Union for Conservation of Nature (IUCN) Red List of Threatened Species released in 2023, over 44,000 species worldwide are facing extinction, with amphibians and mammals severely threatened—at 41% and 26% of species, respectively. In China, the List of National Key Protected Wild Animals includes 980 species, among which 234 are classified as first-class protected species, such as the giant panda and Amur tiger [2]. Traditional wildlife monitoring methods, which rely on manual patrolling and fixed-point observations, suffer from poor timeliness, high labor costs, and limited spatial coverage [3]. Moreover, frequent human presence may disrupt wildlife behavior. The development of non-invasive intelligent monitoring technologies has thus become an urgent scientific challenge in the field of wildlife conservation [4,5].

A variety of technologies have been developed for wildlife monitoring, primarily including non-invasive monitoring, remote sensing, and biotelemetry. Passive infrared camera traps have become a mainstream tool in biodiversity monitoring due to their non-invasive nature, adaptability to diverse terrains, and capability for all-weather, continuous surveillance [6,7]. For instance, SnapShot Safari, the world’s largest camera trap database, has collected over 50 million wildlife images [8], while a single device can generate up to 2 TB of data annually [9]. However, large-scale data processing presents two major challenges: (1) Manual annotation is labor-intensive. For example, the Smithsonian Institution’s 2000 camera traps produce 8.6 million images annually, requiring an estimated 300 person-years of effort for labeling [10]. (2) Visual interpretation is susceptible to errors, with even expert identification exhibiting error rates of 15–20% [11]. These challenges underscore the limitations of relying solely on human effort for data analysis, particularly as image volumes grow exponentially. Consequently, there is a pressing need for automated, intelligent detection algorithms that can efficiently process vast datasets with high accuracy and consistency, thereby reducing both human workload and error rates [12,13].

In recent years, deep learning technologies, particularly convolutional neural networks (CNNs), have significantly advanced automated wildlife image recognition [14]. Compared to traditional methods based on handcrafted features such as HOG and SIFT, CNNs demonstrate greater robustness in complex environments through end-to-end feature learning [15,16]. CNN-based models achieve automated recognition through powerful feature extraction capabilities, thereby reducing time and cost associated with large-scale image processing [17]. Despite these advantages, CNN-based methods face several practical challenges, such as multi-class recognition, background clutter, and imbalanced class distributions, which often degrade model performance. To address these challenges, researchers have proposed various improvements, including two-stage recognition systems [18], multi-channel network architectures [19], and empty-frame optimization strategies [20].

With the evolution of object detection architectures, two-stage detectors have been widely adopted for fine-grained classification tasks due to their high accuracy. In [21], Faster R-CNN was combined with InceptionResNet V2, leveraging the latter’s deep feature extraction capabilities to classify ten European wild mammal species. In [22], a three-stage Faster R-CNN system was developed, enabling fully automated video-to-population assessment workflows. However, the high computational cost and slower inference speed of two-stage detectors make them less suitable for large-scale or real-time wildlife monitoring, particularly in resource-constrained scenarios. Therefore, researchers have begun to explore methods to accelerate detection under limited computational resources, aiming to overcome the inefficiency of two-stage detectors during inference. One mainstream approach focuses on improving and optimizing single-stage detectors to enhance detection accuracy and maintain fast speed to achieve a balance between accuracy and efficiency [23]. Recent advances in single-stage models, particularly the You Only Look Once (YOLO) series, have demonstrated remarkable performance in wildlife detection tasks. In [24], a comprehensive evaluation of YOLOv5, Cascade R-CNN, and FCOS was conducted on the Northeast China Tiger and Leopard National Park dataset, concluding that YOLOv5m achieved the best overall performance. WD-YOLO [25] introduced weighted multi-scale feature fusion to better detect animals of varying sizes. In [26], WilDect-YOLO was proposed, integrating residual CSPDarknet53, DenseNet modules, and an improved SPP-PANet architecture [27,28] to improve detection in cluttered natural environments. In [29], TMS-YOLO was developed by embedding attention mechanisms, achieving more accurate detection on both a custom dataset and the Turkish wildlife dataset.

Driven by the demand for real-time ecological monitoring, lightweight detection models are particularly crucial for camera traps. In practice, conventional camera traps suffer from significant information latency, as data retrieval is often required only after several months or even a year, making it difficult to provide timely monitoring data on protected wildlife. Consequently, the development of smart camera traps equipped with on-device lightweight detection algorithms has emerged as a key research direction. However, due to the inherent challenges in studying endangered species, research in this direction remains limited. For instance, models such as MCFP-YOLO [30], RTAD [31], and YOLO-SAG [32] have achieved impressive efficiency in animal detection. Nevertheless, these models were trained on common species with abundant and easily accessible data and thus may not generalize well to the unique challenges of protected wildlife detection. Conversely, studies such as WilDect-YOLO [26] and WildARe-YOLO [33] have explored endangered species detection, but their datasets were mainly composed of images collected from the Internet, which do not reflect the conditions of passive, in situ field monitoring. As a result, there remains a gap between existing lightweight object detection models and their practical deployment for protected wildlife monitoring in real-world ecological settings.

To bridge the gap, we propose YOLO11-APS, a lightweight object detection model tailored for the efficient and accurate monitoring of protected wildlife through camera trap images. Based on YOLO11, YOLO11-APS integrates several efficient modules: the self-Attention and Convolution (ACmix) module to enhance global and local feature extraction, the Partial Convolution (PConv) module to reduce parameters and accelerate inference, and the SlimNeck paradigm to improve multi-scale feature fusion. The primary goal of this study is to optimize detection algorithms for deployment on terminal devices, thereby contributing to intelligent and automated ecological monitoring. The development of smart camera traps holds significant potential. The advancement of specialized models such as YOLO11-APS not only supports the monitoring of protected wildlife but also provides a theoretical and practical foundation for the intelligent evolution of camera traps.

The main contributions of this study are as follows:

- We construct a dataset of protected wildlife from camera traps, encompassing diverse scenarios, varying object scales, and complex backgrounds. This dataset addresses the scarcity of specialized data and provides a valuable resource for ecological monitoring research.
- We propose YOLO11-APS, an enhanced lightweight model built on YOLO11n. By integrating efficient components such as ACmix, PConv, and SlimNeck, our model achieves an optimal balance between accuracy and computational efficiency, improving feature representation and inference speed to facilitate deployment on ecological monitoring resource-constrained devices.
- Experimental results demonstrate that YOLO11-APS outperforms mainstream object detection models in both accuracy and efficiency on protected wildlife data, underscoring its potential as a deployable solution for intelligent, real-time monitoring in conservation areas.

## 2. Materials and Methods

### 2.1. Data Acquisition

The data for this study were collected through continuous monitoring in Baishanzu National Park (27°32′–27°58′ N, 118°58′–119°22′ E), Lishui City, Zhejiang Province, China, from 2015 to 2020. The data were captured using Hikvision network cameras (Hikvision Digital Technology Co., Ltd., Hangzhou, China). The detailed technical specifications of the devices are provided in Table 1. The cameras operated via an infrared-triggered mechanism, capturing three consecutive still images within one second of activation and then recording a 10 s video at 30 frames per second (FPS). The images and videos had original resolutions of 4000 × 3000 and 1440 × 1080 pixels, respectively, and were saved in JPG and MP4 formats. To make full use of the video data, frames were manually extracted from the videos at a rate of three frames per second and saved as JPG images.

**Figure 1:**
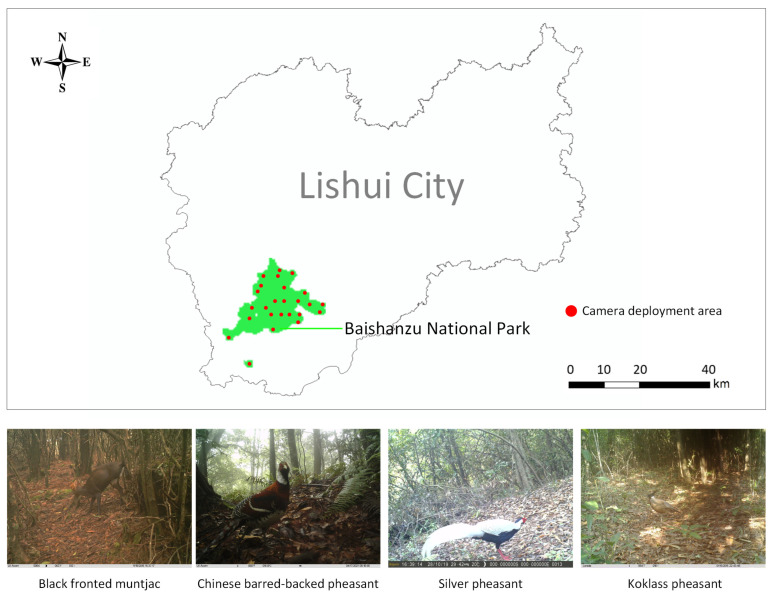
Study area and representative protected wildlife species in the region.

Although image quality from the continuously operating camera traps was sometimes affected by adverse weather conditions (e.g., rainfall, haze) or changing illumination (e.g., sunny, cloudy, rainy, nighttime), the devices reliably recorded data, ensuring dataset completeness. Cameras were installed at heights ranging from 20 to 200 cm above the ground, adjusted according to the local terrain and the target species. The trigger distance of the cameras ranged from 10 to 15 m. Deployment points were strategically selected based on animal activity patterns and habitat features, prioritizing trails, water sources, and foraging areas, while avoiding direct sunlight or exposure to wind and rain, and ensuring sufficient spacing to cover distinct habitats. The spatial distribution of the camera deployment sites is illustrated in Figure 1.

This study focused on four species from the List of Key Protected Wild Animals in China, selected for their relatively abundant image samples, which support effective model training and analysis. The selected species are black fronted muntjac (*Muntiacus crinifrons*), Chinese barred-backed pheasant (*Syrmaticus ellioti*), silver pheasant (*Lophura nycthemera*), and koklass pheasant (*Pucrasia macrolopha*), all of which are representative of the local ecosystem. According to the IUCN Red List, the black-fronted muntjac is classified as Vulnerable (VU) and the Chinese barred-backed pheasant as Near Threatened (NT). Images were excluded if they were blurry, obstructed, poorly illuminated, highly redundant across video frames, or did not contain the target species. After filtering, the final dataset comprised 1603 images: 355 of black fronted muntjac, 304 of Chinese barred-backed pheasant, 544 of silver pheasant, and 400 of koklass pheasant. Figure 1 illustrates the study area and representative protected wildlife species in the region.

### 2.2. Data Preprocessing

Images were manually annotated using the LabelImg tool. Subsequently, the dataset was partitioned into training, validation, and test sets at an 8:1:1 ratio using a stratified sampling strategy to ensure balanced representation of each class across subsets.

**Figure 2:**
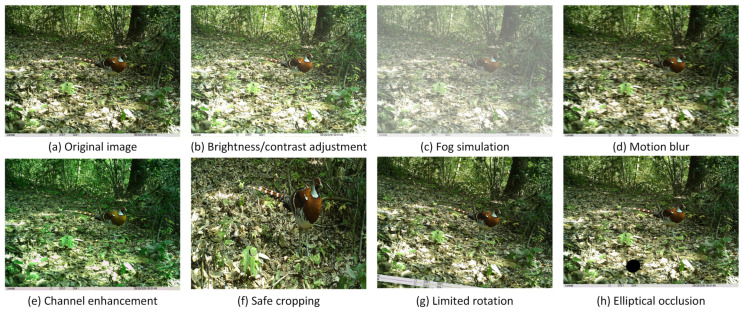
Example of data augmentation effects.

To enhance the model’s generalization capability and robustness while mitigating overfitting during training, a series of data augmentation strategies were employed. These strategies included brightness and contrast adjustment, fog simulation, motion blur, channel enhancement, safe cropping, limited rotation, and elliptical occlusion. These techniques were designed to simulate real-world scenarios, including changes in lighting conditions, foggy weather, animal movement, color variations, partial occlusions, camera misalignment, and environmental obstructions. Figure 2 represents the examples of the augmented images. This augmentation expanded the training set to 10,248 images.

### 2.3. Overview of Baseline Model

YOLO11 was selected as the baseline model for this study due to its superior accuracy, efficiency, and broad adaptability in real-world applications. In terms of architectural design, YOLO11 introduces several optimizations to both the backbone and neck components. In the backbone, the cross-stage partial with spatial attention (C2PSA) module is incorporated, while the original cross-stage partial focus (C2f) module is replaced with the C3k2 module. The C3k2 module enhances model flexibility by allowing customization of the convolutional block size. The C2PSA module integrates a position-sensitive attention (PSA) mechanism, which combines multi-head attention (MHA) and a feedforward neural network (FFN) to further improve feature extraction capabilities. Additionally, the C2PSA module optionally incorporates shortcuts to optimize gradient propagation and facilitate training. For the neck, YOLO11 adopts a path aggregation network (PAN) [28] structure with the C3k2 module embedded, which aids in aggregating multi-scale features and optimizing feature transmission. The Head of YOLO11 is similar to that of YOLOv8 but introduces depthwise convolution (DWConv) [34] in the classification branch to reduce redundant computations and improve efficiency. Regarding the loss function, binary cross-entropy loss (BCE) is employed for classification, while the regression branch combines distribution focal loss (DFL) with complete IoU loss (CIoU). The weighted integration of these three losses enables YOLO11 to achieve higher accuracy in object detection.

### 2.4. Proposed Model

**Figure 3:**
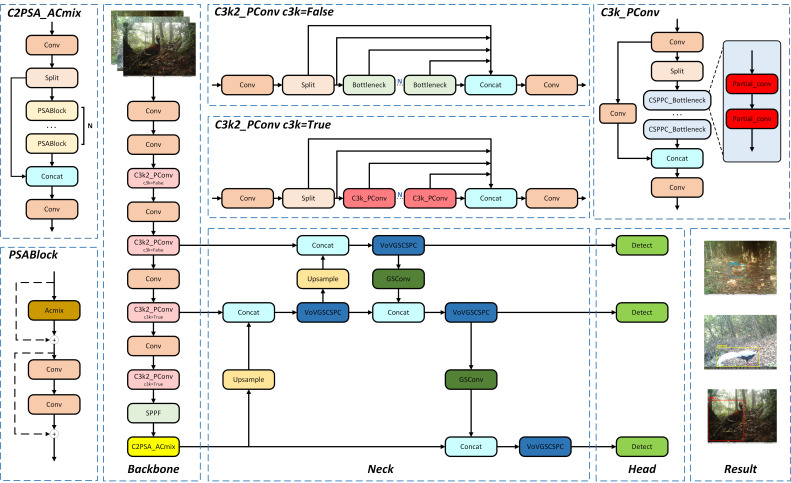
Architecture of our proposed YOLO11-APS.

This study proposes the YOLO11-APS model illustrated in Figure 3, which introduces three key improvements based on the YOLO11n architecture. (1) The ACmix module is integrated into the C2PSA module to enhance the model’s ability to capture global contextual information by leveraging the complementary strengths of global attention and local convolution. (2) To address the computational overhead of the dynamic convolution in the C3k2 module, the lightweight PConv module is employed as a replacement, streamlining the feature extraction process. (3) The neck adopts the SlimNeck paradigm, which combines GSConv and VoV-GSCSPC structures to enhance multi-scale feature representation while reducing the number of model parameters. The head structure and loss function remain consistent with those of the baseline model.

#### 2.4.1. Self-Attention and Convolution

In this study, the MHA is replaced by the ACmix module [35] to achieve an effective fusion of global perception and local feature extraction. The ACmix module integrates the advantages of both self-attention and convolution operations, enhancing local feature modeling while reducing computational complexity. This improvement enables the model to more effectively capture fine-grained discriminative information.

**Figure 4:**
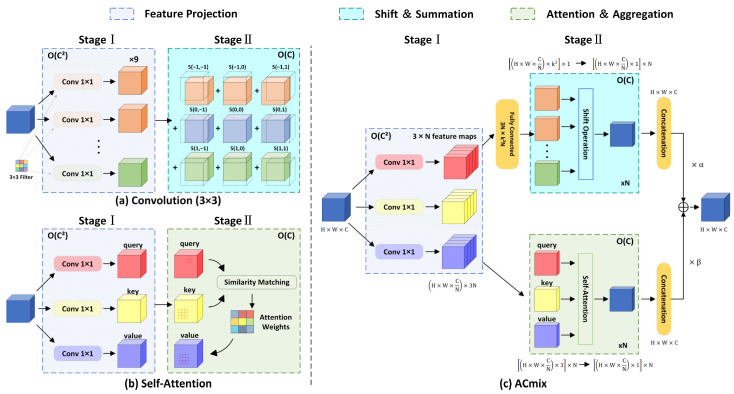
Overview of the ACmix structure, integrating convolution and self-attention in a unified module.

The hybrid architecture of ACmix is illustrated in Figure 4, where Figure 4a illustrates that the output of a 3 × 3 convolution can be decomposed into the summation of several translated feature maps. Each of these maps is generated by applying a 1 × 1 convolution to a specific spatial kernel weight. Figure 4b demonstrates the self-attention process, where the input feature map is first transformed into query, key, and value tensors via 1 × 1 convolutions. Subsequently, to aggregate the values, the attention weights are computed from the similarity between the query and key. Figure 4c combines the characteristics of both approaches. Initially, three parallel 1 × 1 convolutions project the input features. After that, the resulting intermediate features are processed through two separate branches—one for convolution and one for self-attention—whose outputs are summed to produce the final result. The computational complexity for each unit is annotated in the upper-right corner of the figure. By sharing 1 × 1 convolutions and integrating convolutional and self-attention operations, ACmix reduces computational overhead and optimizes channel-wise complexity.

The core of the ACmix architecture lies in its mathematical reformulation, which unifies self-attention and convolution into an equivalent computational paradigm. Given an input feature map $X\in R^{H\times W\times C}$, where H, W, and C denote the height, width, and number of channels, respectively, an intermediate feature representation $V=XW_{v}$ is first generated via a shared 1 × 1 convolution. Here, $W_{v}\in R^{C\times d}$ is the projection matrix and d denotes the dimensionality of the projected feature space. In the self-attention branch, the dynamic attention weights $A_{\mathrm{att}}$ are derived from the query and key matrices. The query matrix is $Q=XW_{q}\in R^{N\times d}$ and the key matrix is $K=XW_{k}\in R^{N\times d}$, where $W_{q}$, $W_{k}\in R^{C\times d}$ are learnable projection matrices, and N=H×W is the number of spatial positions. Subsequently, the attention matrix is computed as:

(1) $$A_{\mathrm{att}}=\mathrm{Softmax}\left(\frac{QK^{⊤}}{\sqrt{d}}\right)$$

In the convolution branch, static kernel functions are parameterized via positional encoding, reconstructing the convolution operation as a sparse attention matrix $A_{\mathrm{conv}}\in R^{N\times N}$, where each element satisfies:

(2) $$A_{\mathrm{conv}}\left(i,j\right)=\sum_{k}\omega_{k}\cdot \delta \left(\left\lfloor j/S\right\rfloor -i+p_{k}\right)$$

Here, $\omega_{k}$ denotes the learnable convolutional kernel weights, $p_{k}$ represents the predefined offset, $\delta \left(\cdot \right)$ is the Kronecker delta function, and S is the spatial width of the feature map. By unifying both operations into a matrix multiplication form Y=A ⋅V, ACmix enables dual-branch feature fusion through a composite coefficient λ:

(3) $$Y_{\mathrm{out}}=\lambda \cdot A_{\mathrm{att}}V+\left(1-\lambda \right)\cdot A_{\mathrm{conv}}V$$

This architecture retains the global interaction modeling capability of the self-attention mechanism with a complexity of $O\left(N^{2}d\right)$, where N=H ×W is the total number of spatial positions and d is the channel dimension. By employing a shared-weight projection matrix $W_{v}$, the overall computational complexity is reduced from the conventional additive form $O\left(N^{2}d+Nk^{2}d\right)$ to $O\left(Nd^{2}\right)$. This approach effectively balances the trade-off between receptive field size and computational efficiency.

#### 2.4.2. Partial Convolution

**Figure 5:**
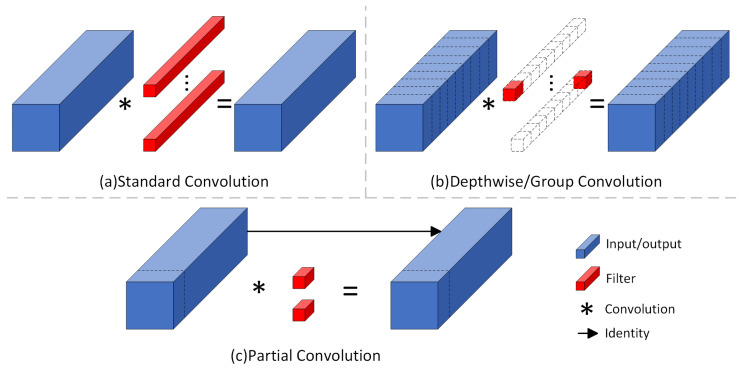
Comparison of convolutional structures.

To achieve model lightweighting, this study replaces the C3k2 module in the Backbone with the PConv module. PConv is an efficient computational optimization strategy designed to reduce redundant operations and enhance the computational efficiency of neural networks [36]. As illustrated in Figure 5, the core idea of PConv is to perform convolution only on a subset of channels while keeping the remaining channels unchanged. This approach lowers the overall computational cost and preserves essential feature information. Compared to traditional convolution, PConv reduces redundancy without compromising performance, thereby boosting inference speed and decreasing model parameters. This makes it especially suitable for resource-constrained applications.

Specifically, for an input feature map $I\in R^{c\times h\times w}$, PConv applies a k×k convolution only to the first $c_{p}=c/4$ channels. The resulting floating-point operations (FLOPs) are $h\times w\times k^{2}\times c_{p}^{2}$, which is only 1/16 of the FLOPs required by a standard convolution (SC) when $c_{p}=c/4$. Additionally, the memory access volume of PConv is reduced to $h\times w\times 2c_{p}$, approximately 1/4 that of SC, thereby alleviating the I/O bottleneck. To fully exploit information from all channels, PConv is typically combined with pointwise convolution [34], forming a T-shaped receptive field computation pattern. This allows the computation ratio across channels to be dynamically adjusted based on task requirements, overcoming the limitations of fixed channel partitioning in C3k2 modules. The work [36] showed that PConv significantly improves inference speed and maintains relatively low FLOPs, demonstrating superior performance across GPU, CPU, and ARM processors.

#### 2.4.3. SlimNeck

In this study, the SlimNeck paradigm [37] is introduced in the neck part of the model, where GSConv replaces convolution (Conv), and VoV-GSCSPC replaces the C3k2 module in the Neck. Conv is widely used in YOLO models to effectively capture spatial information through the linear combination of local receptive fields. However, its dense parameters and high computational cost limit the model’s lightweight potential [38]. GSConv combines the advantages of SC and depth-wise separable convolution (DSC) [39] by employing a feature-mixing strategy that integrates SC features into the output of DSC. A channel shuffle operation is used to make the DSC output more similar to that of SC, thus improving feature representation and maintaining low computational cost. The VoV-GSCSPC module further optimizes the feature fusion process, reducing both computational burden and network complexity while maintaining sufficient accuracy.

**Figure 6:**
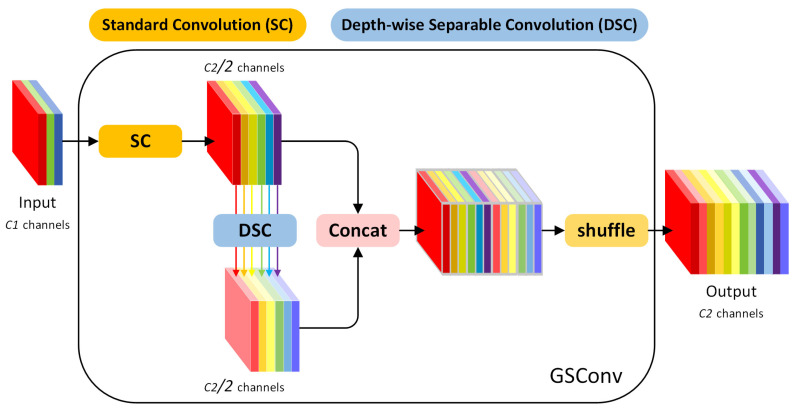
The structure of GSConv.

Specifically, GSConv is a lightweight convolutional design aimed at balancing computational efficiency and feature extraction capability. As illustrated in Figure 6, GSConv consists of two stages: the input feature map is processed by an SC to generate intermediate features occupying half of the output channels; these intermediate features are further processed using a DSC. The outputs from both paths are then concatenated and mixed through a channel shuffle operation, ensuring complete information interaction. This design significantly reduces the computational complexity (approximately 50% of SC) and preserves feature expressiveness close to that of SC. GSConv is commonly adopted in neural network architectures designed for mobile and edge devices to improve runtime efficiency.

**Figure 7:**
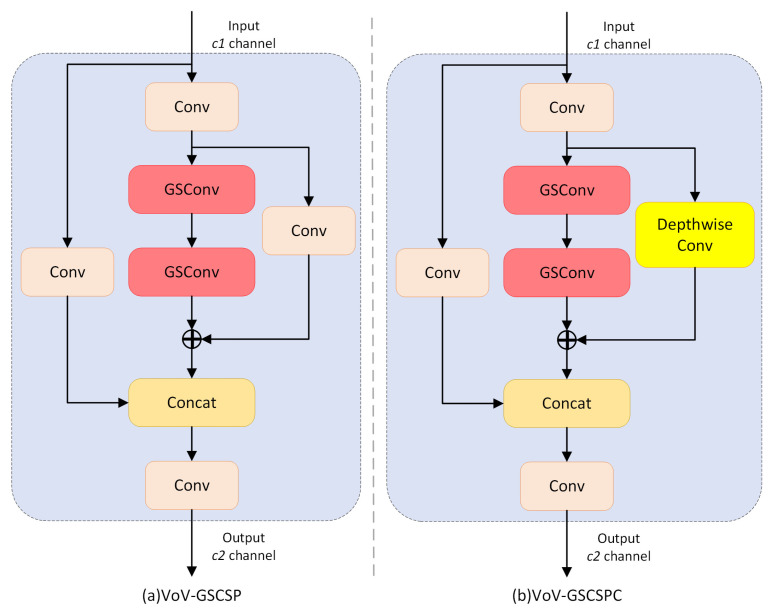
The structure of VoV-GSCSP and VoV-GSCSPC.

VoV-GSCSP is a cross-stage partial network module built upon GSConv, which further optimizes the balance between computational cost and feature fusion efficiency. It incorporates the “one-shot aggregation” concept from VoVNet [40]. In its implementation, VoV-GSCSP splits the input features into two branches: one retains the original information via a shortcut connection, and the other undergoes multi-level feature extraction and fusion through GSConv. The advantage of VoV-GSCSP lies in its ability to reduce computational burden in the neck (with FLOPs decreased by an average of 15.72%) while simultaneously enhancing detection accuracy through dense feature interaction. This study adopts a lightweight variant of VoV-GSCSP, named VoV-GSCSPC, which fixes the number of hidden channels to half of the output channels, further reducing computational cost. Additionally, DWConv is applied to compress the parameter size. The structures of VoV-GSCSP and VoV-GSCSPC are illustrated in Figure 7.

### 2.5. Experimental Setup

All experiments were conducted on a workstation equipped with an Intel Core i5-13600KF CPU (14 cores, 3.5 GHz), an NVIDIA RTX 4060 Ti GPU (16 GB), and 32 GB RAM. The software environment was configured with Python 3.9.7, PyTorch 1.12.1, CUDA 12.3, and cuDNN 8.3.2. All models were initialized with pretrained weights and trained for up to 300 epochs. The training was performed with a batch size of 8, an input size of 640 × 640, and an initial learning rate of 0.01. SGD optimizer was used with momentum 0.937 and weight decay 0.005.

### 2.6. Evaluation Metrics

To evaluate the model’s performance, this study employs two categories of metrics: detection performance and model complexity.

Detection performance is primarily assessed using precision (P), recall (R), and mean average precision (mAP). P and R are calculated based on true positives (TP), false positives (FP), true negatives (TN), and false negatives (FN). As a standard metric, mAP evaluates the overall detection capability of a model, where higher values indicate better performance. Formally, mAP is the mean of the average precision (AP) across all object categories, where AP is calculated as the area under the corresponding P-R curve. These metrics are calculated as follows:

(4) $$P=\frac{TP}{TP+FP}$$

(5) $$R=\frac{TP}{TP+FN}$$

(6) $$AP=\int_{0}^{1}P\left(R\right)dR$$

(7) $$mAP=\frac{\sum_{n=1}^{N}AP_{n}}{N}$$

where N denotes the number of classes.

For model complexity, three key metrics are considered: the number of parameters (Params), FLOPs, and model size. FLOPs quantify the total number of floating-point operations performed by the model. Params reflect the storage and computational resources required by the model.

## 3. Results

### 3.1. Class-Wise Detection Performance

**Figure 8:**
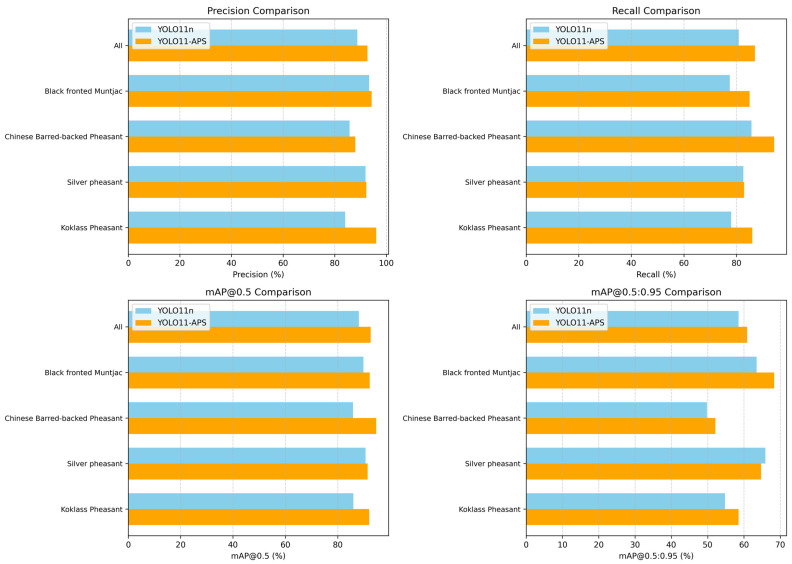
Per-class detection performance comparison between YOLO11n and YOLO11-APS.

Figure 8 compares the detection performance of the proposed model against the baseline model across different species. The results indicate that, on the average category (All), the proposed model achieves improvements of 3.9%, 6.1%, 4.5%, and 2.4% in precision, recall, mAP@0.5, and mAP@0.5:0.95, respectively. The proposed model demonstrates superior detection accuracy across all four species. Specifically, the mAP@0.5 for the black-fronted muntjac, Chinese barred-backed pheasant, silver pheasant, and koklass pheasant increased by 2.4%, 9.9%, 0.8%, and 6.0%, respectively. These results demonstrate that the proposed model possesses stronger feature extraction capabilities, particularly for uncommon protected wildlife species.

### 3.2. Ablation Experiments

#### 3.2.1. Effect of Improvement Modules

To explore the underlying mechanisms of the proposed model, we designed eight ablation experiments to quantify the impact of each module and their combinations on overall detection performance and model complexity. The experimental configurations are designed as follows: Exp 1 serves as the baseline model. Exp 2–4 individually incorporate the ACmix, PConv, and SlimNeck modules, respectively. Exp 5–7 represent two-module combinations, specifically ‘ACmix + PConv’, ‘ACmix + SlimNeck’, and ‘PConv + SlimNeck’. Exp 8 integrates all three modules, constituting the final proposed model. The results of the ablation experiments are presented in Table 2. The ACmix mechanism enhanced the model’s ability to extract local fine-grained features, resulting in a 0.3% improvement in mAP@0.5. The PConv module effectively reduced redundant features, thereby alleviating computational burden and memory overhead while maintaining strong spatial feature extraction. This led to a 0.4% increase in mAP@0.5, along with reductions of 0.31 M parameters, 0.3 GFLOPs, and 0.63 MB in model size. The SlimNeck structure improved feature transmission efficiency and overall network performance, achieving a 1.5% gain in mAP@0.5, while reducing Params by 0.1 M, GFLOPs by 0.5, and model size by 0.2 MB. Evaluating the dual-module combinations revealed that each configuration achieved additional performance improvements compared to the baseline, highlighting the complementary effects of different modules. Finally, the integration of all three modules into the proposed YOLO11-APS architecture yielded the most significant results. Compared to the baseline, it achieved gains of 3.8%, 6.3%, 4.5%, and 3.7% in precision, recall, mAP@0.5, and mAP@0.5:0.95, respectively. Concurrently, the model complexity was reduced, with decreases of 0.26 M in parameters, 0.7 GFLOPs in computation, and 0.5 MB in size.

#### 3.2.2. Attention Mechanisms Comparison Study

We conducted a comparative study on various attention mechanisms incorporated into the YOLO11 architecture, including MLCA [41], DLKA [42], DAT [43], iRMB [44], Triplet Attention [45], and ACmix. As summarized in Table 3, while ACmix does not achieve the best results in terms of either detection performance or model complexity, it represents the optimal balance between the two.

#### 3.2.3. Convolutional Modules Comparison Study

To investigate the influence of different convolutional modules, we evaluated WTConv [46], CG Block [47], LDConv [48], HetConv [49], UIB [50], and PConv. As shown in Table 4, although PConv does not achieve the highest precision, recall, or mAP@0.5, it exhibits favorable lightweight performance. Therefore, considering both accuracy and computational efficiency, PConv emerges as a more suitable choice for lightweight model design.

#### 3.2.4. Neck Structures Comparison Study

We further investigated the impact of different neck structures, including CCFF [51], RepGFPN [52], FreqFusion [53], and SlimNeck. As shown in Table 5, while CCFF achieves the best lightweight performance, its mAP@0.5 and mAP@0.5:0.95 are relatively low. In contrast, SlimNeck offers the best trade-off between detection performance and model complexity. It enhances multi-scale feature fusion while simultaneously achieving model lightweighting.

### 3.3. Contrast Experiments

To comprehensively evaluate the overall performance of the proposed model, we selected 11 mainstream object detection models for comparison. The detailed experimental results are presented in Table 6. As shown, YOLO11-APS outperforms all compared models across four key metrics: precision, recall, mAP@0.5, and mAP@0.5:0.95, demonstrating superior detection capability. In terms of model complexity, YOLO11-APS achieves 2.32 M Params, 5.6 GFLOPs, and a model size of 4.75 MB, ranking second only to YOLOv5n. The proposed model achieves high detection accuracy while reducing the demand for storage and computational resources. In summary, YOLO11-APS achieves an optimal trade-off between detection accuracy and model complexity, offering a reliable and practical solution for protected wildlife identification in camera trap applications.

### 3.4. Visualized Analysis

**Figure 9:**
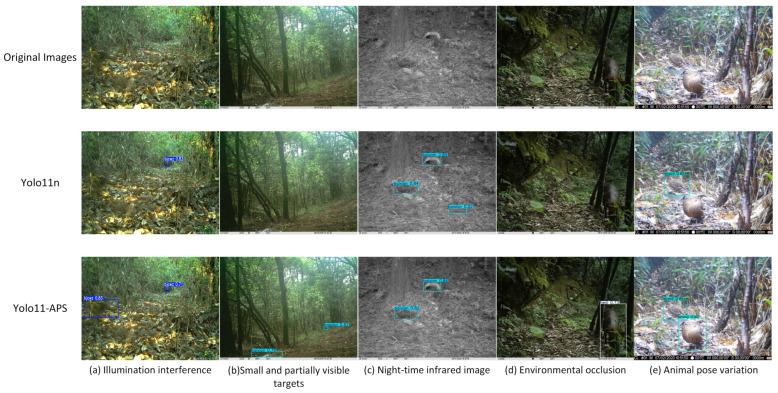
Qualitative comparison of YOLO11n-APS with YOLO11 on the custom dataset.

Figure 9 presents a set of illustrative detection results to visually compare the performance of the baseline YOLO11n model with the proposed YOLO11-APS model under various challenging conditions commonly encountered in natural environments. The selected images cover scenarios such as illumination interference, small and partially visible targets, night-time infrared imaging, environmental occlusion, and animal pose variation. As shown, YOLO11-APS demonstrates superior detection stability and robustness under these challenging conditions, effectively reducing both false positives and missed detections to achieve more accurate target recognition.

**Figure 10:**
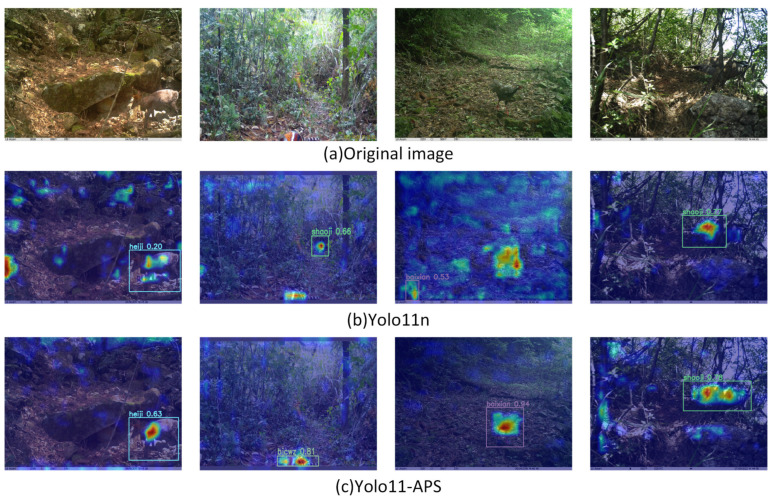
Comparison of heatmaps between YOLO11n and YOLO11-APS.

We employed the Grad-CAM method to generate the heatmaps for model interpretability. Figure 10 presents a comparison of these heatmaps between the baseline and our proposed model. In these visualizations, the redder regions indicate areas where the model’s attention is more concentrated. The more focused and better aligned these red areas are with the target, the more precise the model’s attention and the more effective its feature extraction capabilities become. As observed, compared to the baseline, our proposed model demonstrates superior performance by significantly enhancing its focus on target regions, which in turn improves recognition accuracy.

### 3.5. Cross-Dataset Model Validation

**Figure 11:**
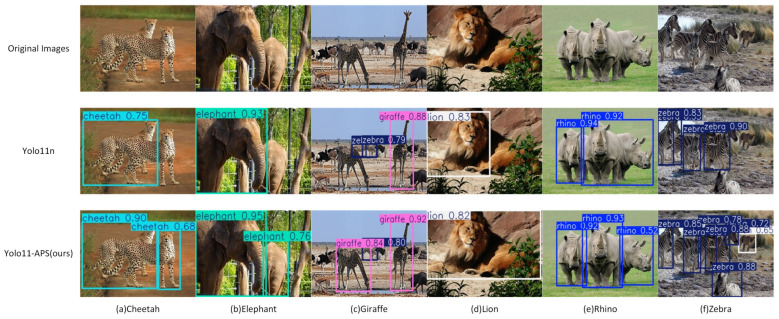
Detection comparison between YOLO11n and YOLO11-APS on the WAFE dataset.

To validate the effectiveness of YOLO11-APS beyond our custom dataset, this study conducted experiments on the publicly available Wild Animal Facing Extinction (WAFE) dataset [54] which focuses on common animals in danger. The dataset comprises 7634 images across six categories: rhinoceros (1389), cheetah (1150), lion (1277), elephant (1288), giraffe (1209), and zebra (1321). It was partitioned into training, validation, and test sets in an 8:1:1 ratio. Data augmentation was applied to the training set to enhance model robustness. Experimental settings were consistent with those of Section 2.5. As shown in Table 7, our proposed YOLO11-APS model achieved mAP@0.5 of 0.3% and mAP@0.5:0.95 of 0.2% higher than the baseline. This further demonstrates its robustness and transferability to unseen datasets. Corresponding visualization results are presented in Figure 11.

## 4. Discussion

The proposed YOLO11-APS represents a methodological innovation and exemplifies the deep integration of artificial intelligence with critical ecological conservation needs. By strategically incorporating modules such as ACmix, PConv, and SlimNeck, the model maintains high detection accuracy in complex natural environments while significantly reducing computational costs. This optimal balance between accuracy and efficiency offers a practical solution for deploying intelligent camera traps in resource-constrained environments.

A key challenge in wildlife conservation is the timely and accurate assessment of population dynamics and distribution patterns for endangered species [55]. However, traditional methods such as field surveys and post hoc image screening are often hampered by data scarcity and complex monitoring conditions, rendering them labor-intensive and error-prone [6]. YOLO11-APS directly addresses these limitations by enabling rapid automatic identification of target species in large volumes of camera trap images. This capability effectively reduces empty frames and misclassifications, thereby improving monitoring efficiency and providing more objective, systematic data to support conservation strategies for endangered wildlife.

Furthermore, the model’s robustness in complex natural environments enables it to effectively cope with challenges such as variable illumination, vegetation occlusion, diverse animal postures, and high background similarity. This resilience ensures that YOLO11-APS maintains stable performance across different seasons and ecosystems, establishing a reliable foundation for long-term and continuous population monitoring. By facilitating accurate identification and classification of endangered species, the model enables the acquisition of detailed data on population dynamics, including distribution ranges, abundance changes, and behavioral patterns. Such information is crucial for evaluating conservation effectiveness, formulating adaptive ecological management strategies, and detecting potential threats such as invasive species or disease outbreaks.

The lightweight design of YOLO11-APS paves the way for large-scale, networked monitoring systems. Its capability for deployment on low-power edge devices enables truly distributed monitoring, eliminating the reliance on high-performance servers. This is particularly crucial for monitoring extensive habitats and enhancing the frequency and timeliness of data collection. Combined with complementary technologies such as geographic information systems (GIS), drone imagery, and long-term ecological databases, YOLO11-APS has the potential to serve as the technological core of a multi-scale framework for ecological research and conservation decision-making.

Moreover, the powerful feature extraction capabilities of YOLO11-APS can profoundly advance ecological and behavioral studies, such as analyzing animal foraging, migration, and activity patterns, thereby enriching the understanding of species’ ecological habits and habitat requirements. This AI-driven monitoring paradigm not only reduces human labor costs but also enables data-driven conservation decisions, providing critical technical support for sustainable ecosystem management.

Transferability tests on diverse protected wildlife datasets demonstrate the robustness and adaptability of YOLO11-APS. This capability is invaluable for cross-regional species monitoring and long-term ecological observation, positioning the model as a general-purpose monitoring tool applicable to diverse ecosystems and species. As AI-driven ecological research evolves, YOLO11-APS is poised for integration into intelligent camera traps, enabling on-device processing and real-time filtering. This integration promises to significantly enhance data acquisition efficiency and promote a paradigm shift in ecological monitoring from passive data accumulation to positive, data-driven decision-making.

Despite these encouraging results, several limitations remain. The custom dataset includes a limited number of protected wildlife species, which may restrict the model’s generalization ability across broader ecological scenarios. In addition, current studies have primarily emphasized algorithmic performance while practical considerations for large-scale ecological deployment remain underexplored. To address these limitations, our future work will proceed in two directions. First, we aim to leverage model outputs to conduct preliminary analyses of species activity patterns, thereby optimizing camera deployment strategies to improve data collection efficiency and enrich protected wildlife image databases. Second, we plan to develop intelligent camera traps that integrate our proposed model, enabling real-world application and iterative refinement through deployment feedback. These continuous efforts aim to drive methodological innovations in lightweight detection models and foster their practical applications in digital ecological conservation.

## 5. Conclusions

This study proposed a lightweight object detection model, YOLO11-APS, tailored for the detection of protected wildlife in camera trap images. By integrating the ACmix module, PConv module, and SlimNeck paradigm into YOLO11n, our model achieves a more efficient feature extraction process while maintaining low computational cost. Experimental results demonstrated that YOLO11-APS significantly outperforms the baseline YOLO11n, achieving precision of 92.7%, recall of 87.0%, mAP@0.5 of 92.6%, and mAP@0.5:0.95 of 62.2%. Meanwhile, the model reduces its size, parameters, and FLOPs by 9.5%, 10.1%, and 11.1%, respectively, showcasing an optimal balance between accuracy and efficiency. Ablation studies further verify the effectiveness of each enhanced component and confirm their complementary contributions to overall performance. Furthermore, evaluation on an unseen wildlife dataset demonstrates the model’s strong transferability and robustness in real-world scenarios. In conclusion, YOLO11-APS offers an efficient and reliable solution for automated wildlife detection, with significant potential to advance practical ecological monitoring and conservation applications. Furthermore, future work will focus on expanding the range of species and facilitating real-world deployment of the technology.

## Figures and Tables

**Table 1:** Technical parameters of the camera trap.

Parameter	Description
Camera Name	Hikvision Network Camera
Model	DS-2XS6C8FZC-QYBS (6 mm)
Sensor	1/1.8′′ Progressive Scan CMOS
Infrared Mode	Low-glow IR (850 nm)
Field of View	Horizontal 50° ± 5°, Vertical 20° ± 5°
Detection Range	Up to 15 m
Trigger Speed	<0.3 s

**Table 2:** Results of ablation experiments.

Exp	Configuration	P (%)	R (%)	mAP@0.5 (%)	mAP@0.5:0.95 (%)	Params (M)	GFLOPs	Model Size (MB)
1	baseline	88.9	80.9	88.1	58.5	2.58	6.3	5.25
2	baseline + ACmix	91	82.4	88.4	58.6	2.59	6.3	5.23
3	baseline + PConv	89	82.5	88.5	59.3	2.27	6	4.62
4	baseline + SlimNeck	90.4	86	89.6	59.6	2.48	5.8	5.05
5	baseline + ACmix + PConv	92	85.1	90.1	61.9	2.28	6.1	4.63
6	baseline + ACmix + SlimNeck	91.2	85.3	91.8	61.9	2.49	5.8	5.1
7	baseline + PConv + SlimNeck	90.4	83.8	91	61.6	2.31	5.6	4.73
8	YOLO11-APS(ALL)	92.7	87	92.6	62.2	2.32	5.6	4.75

**Table 3:** Performance of various attention mechanisms.

Method	P (%)	R (%)	mAP@0.5 (%)	mAP@0.5:0.95 (%)	Params (M)	GFLOPs	Model Size (MB)
YOLOv11 (Baseline)							
+MLCA [41]	90.9	81.6	87.7	58.5	2.53	6.3	5.13
+DLKA [42]	92.3	82.3	88.7	59.5	3.37	6.9	6.73
+DAT [43]	91.3	82	87.8	58.5	2.61	6.9	5.29
+iRMB [44]	93.1	79.7	87.8	59	2.60	8.2	5.26
+Triplet Attention [45]	90.8	81.1	88.2	59.4	2.58	6.4	5.14
+ACmix (ours)	91	82.4	88.4	58.6	2.59	6.3	5.23

**Table 4:** Performance of various convolution modules.

Method	P (%)	R (%)	mAP@0.5 (%)	mAP@0.5:0.95 (%)	Params (M)	GFLOPs	Model Size (MB)
YOLOv11 (Baseline)							
+WTConv [46]	91.8	80.3	88.1	56.6	2.79	7.5	5.6
+CG Block [47]	92.6	83.5	89.6	58.6	2.82	7.1	5.68
+LDConv [48]	90.9	84.1	90.9	59	2.81	7.6	5.65
+HetConv [49]	92.1	82.4	88.5	58.9	2.8	7.5	5.62
+UIB [50]	89.3	78.2	84.7	56	2.83	7.2	5.7
+PConv (ours)	89	82.5	88.5	59.3	2.27	6	4.62

**Table 5:** Performance of various neck structures.

Method	P (%)	R (%)	mAP@0.5 (%)	mAP@0.5:0.95 (%)	Params (M)	GFLOPs	Model Size (MB)
YOLOv11 (Baseline)							
+CCFF [51]	90.9	82.1	87.4	56.1	1.8	5.3	3.74
+RepGFPN [52]	91.5	79.3	84.9	57.9	2.89	6.7	5.83
+FreqFusion [53]	88.7	83.4	87	56.1	2.43	6.8	4.96
+SlimNeck (ours)	90.4	86	89.6	59.6	2.48	5.8	5.05

**Table 6:** Performance comparison of various models on custom datasets.

Model	P (%)	R (%)	mAP@0.5 (%)	mAP@0.5:0.95 (%)	Params (M)	GFLOPs	Model Size (MB)
YOLOv3-tiny	81.4	79.5	81.6	45.1	8.67	12.9	16.6
YOLOv5n	91.1	82	86.8	55.2	1.76	4.1	3.73
YOLOv7	92.3	83.9	89.6	59	6.02	13	11.7
YOLOv8n	89.7	81.9	87.3	59.4	3.01	8.1	5.39
YOLOv10n	86.5	82.5	87.5	57.9	2.7	8.2	5.51
YOLOv12n	91.3	79.1	86.7	57.9	2.56	6.3	5.3
Faster R-CNN	81.8	81.7	86	46.1	28.3	55.87	108
SSD	91.7	85.7	87.1	52.6	24.15	30.58	92.39
Cascade R-CNN	87.9	81.2	83.4	47	56.09	83.67	214
DETR	87.9	86.7	87.5	53.1	41.56	29.23	163
YOLO11n	88.9	80.9	88.1	58.5	2.58	6.3	5.25
YOLO11-APS	92.7	87	92.6	60.9	2.32	5.6	4.75

**Table 7:** Performance comparison between YOLO11n and YOLO11-APS on the WAFE dataset.

Model	P (%)	R (%)	mAP@0.5 (%)	mAP@0.5:0.95 (%)	Params (M)	GFLOPs	Model Size (MB)
YOLO11n	96.4	90	97	85	2.58	6.3	5.25
YOLO11-APS	94	92.7	97.3	85.2	2.32	5.6	4.75

## Data Availability

The data presented in this study are available from the corresponding author upon request. The source code is available as open source at https://github.com/Realworld-D/YOLO11-APS (accessed on 30 November 2025).
